# Low-Cost Plant-Protection Unmanned Ground Vehicle System for Variable Weeding Using Machine Vision

**DOI:** 10.3390/s24041287

**Published:** 2024-02-17

**Authors:** Huangtao Dong, Jianxun Shen, Zhe Yu, Xiangyu Lu, Fei Liu, Wenwen Kong

**Affiliations:** 1 College of Mathematics and Computer Science, Zhejiang A&F University, Hangzhou 311300, China; dhtxxw@163.com (H.D.); 2022611011070@stu.zafu.edu.cn (Z.Y.); 2 Hangzhou Raw Seed Growing Farm, Hangzhou 311115, China; jianxunsh@163.com; 3 College of Biosystems Engineering and Food Science, Zhejiang University, Hangzhou 310058, China; luxyzju@zju.edu.cn (X.L.); fliu@zju.edu.cn (F.L.)

**Keywords:** UGV, machine vision, variable spray, fuzzy rules, PID control, PWM control

## Abstract

This study presents a machine vision-based variable weeding system for plant- protection unmanned ground vehicles (UGVs) to address the issues of pesticide waste and environmental pollution that are readily caused by traditional spraying agricultural machinery. The system utilizes fuzzy rules to achieve adaptive modification of the Kp, Ki, and Kd adjustment parameters of the PID control algorithm and combines them with an interleaved period PWM controller to reduce the impact of nonlinear variations in water pressure on the performance of the system, and to improve the stability and control accuracy of the system. After testing various image threshold segmentation and image graying algorithms, the normalized super green algorithm (2G-R-B) and the fast iterative threshold segmentation method were adopted as the best combination. This combination effectively distinguished between the vegetation and the background, and thus improved the accuracy of the pixel extraction algorithm for vegetation distribution. The results of orthogonal testing by selected four representative spraying duty cycles—25%, 50%, 75%, and 100%—showed that the pressure variation was less than 0.05 MPa, the average spraying error was less than 2%, and the highest error was less than 5% throughout the test. Finally, the performance of the system was comprehensively evaluated through field trials. The evaluation showed that the system was able to adjust the corresponding spraying volume in real time according to the vegetation distribution under the decision-making based on machine vision algorithms, which proved the low cost and effectiveness of the designed variable weed control system.

## 1. Introduction

To ensure consistent and high crop yields in current agricultural production, the application of pesticides is essential for the prevention and treatment of crop pests, diseases, and weeds [1]. However, it is well known that the large-scale use of pesticides and overuse of pesticides have had a gravely negative impact on human health [2,3] and the ecological environment [4,5], making the problem of pesticide residues in agricultural products [6,7] and soil pesticide residues prominent [8,9]. With the continuous development of agriculture, the environmental pollution caused by pesticides can never be ignored [10,11]. Many countries around the world are becoming increasingly concerned about the safety of pesticide use [12], and there is an urgent need for sustainable, low-cost solutions [13,14]. As such, a variety of programs dedicated to reducing pesticide contamination have been proposed [15].

In the process of modernizing agricultural production, due to the equal spraying method employed by the conventional weed spraying equipment, they were unable to make corresponding spraying according to the distribution of vegetation in each plot of the field, thus making it difficult to realize the effective use of pesticides [16]. Therefore, pesticide variable spraying technology is one of the important means to reduce pesticide pollution. The research and promotion of variable spraying technology is of great significance, and its application can reduce the use of conventional pesticide spraying methods in the spraying process caused by a large number of pesticide waste and increase the effective use of pesticides [17].

Future agricultural production will considerably benefit from variable spraying technology since they may significantly reduce the consumption of pesticides used [18,19] and lower the cost of agricultural production processes, as well as effectively control the pesticide residues of farm products exceeding the standard, and improve the quality and safety of agricultural products [20]. At the same time, they will improve agricultural production, increase the income of farmers, and most importantly, reduce pesticide pollution of the agricultural ecological environment, promote green sustainable development, and lead to potential advancements in precision. Additionally, the importance of agriculture is fostering for food safety [21]. To further improve the spraying accuracy based on variable spraying technology, many studies have proposed combining variable spraying technology with other technologies to achieve more accurate on-demand spraying [22]. 

For example, Sheng et al. designed a variable spraying system for plant-protection UAVs based on neural network decision making by combining the current research on variable spraying technology with artificial neural network technology as a basis. Utilizing existing variables, including the environment’s temperature, humidity, wind speed, flight speed, and altitude, a neural network model was developed. Outdoor tests showed that the experimental error was less than 20%, achieving variable spraying in different environments [23]. Nan et al. proposed a contour tracking control method and a variable spraying technique based on CMAC-PID, mainly to control pesticide drift by tracking the contour of the canopy and calculating the corresponding spray flow rate through the volume of the outline and the density of the leaf area, which significantly improved the algorithm’s dynamic tracking performance of the profiling control system, effectively reducing the overall average tracking error and achieving a reduction in costs related to pesticide use and environmental pollution [24]. Liu et al. used a single 3D detection and ranging sensor to sense the fruit trees around the robot and determine the region of interest. ROIs within the point cloud were performed through 2D processing to obtain the center of mass coordinates of the fruit trees and determine the vertical distance of the robot from the rows of fruit trees based on both sides of the FTR using a random sample consensus algorithm. Based on the multi-sensor fusion of the current spray state and the predicted deposition distribution characteristics, the UAV offset and nozzle flow rate of the variable spray system can be obtained. Farm trials showed that the deposition volume error between prediction and trial was within 30%, proving the effectiveness of the system [25]. Liu et al. developed a variable rate spraying system using a deep learning approach to develop new weed classification models and accurately spray on desired weed targets. Three classification CNN (convolutional neural network) models were used, and laboratory and field experiments were conducted to evaluate the spraying performance for weed classification and the accurate spraying of targeted weeds. The results showed that the system reduced pesticide application, ground loss, and air drift, and effectively controlled pesticide pollution of the environment [26]. Overall, although the above studies used a variety of advanced techniques to achieve precision spraying, these techniques may have certain shortcomings in terms of processing speed, which may lead to limitations in practical agricultural applications.

The purpose of this study is to develop and test a machine vision-based variable weeding system for a plant-protection UGV, to adjust the corresponding spraying amount in real time according to the distribution of vegetation under decision making based on machine vision algorithms, and complete real-time on-demand spraying. Combining the variable weeding system with the autonomous path planning function of a UGV and planning the intervals of the UGV’s driving path according to the system’s spraying coverage not only successfully realizes the unmanned variable weeding operation and avoids the problems of leakage and re-spraying that may easily occur in the process of manual spraying, but also provides effective technical support for the realization of the full-area accurate and automatic variable weeding in farmlands.

## 2. Materials and Methods

### 2.1. Design of Variable Weeding System

**Figure 1:**
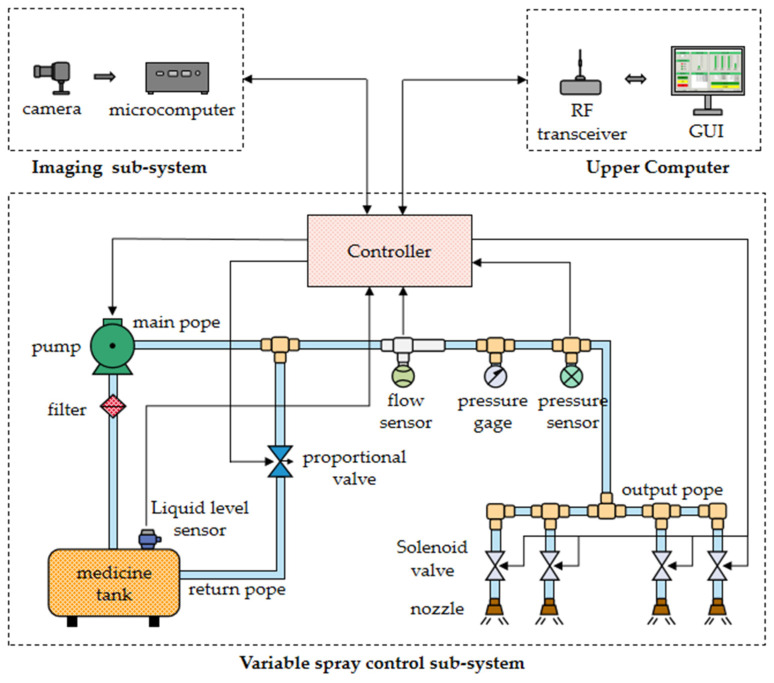
The variable weeding system.

Currently, in the field of agricultural plant protection variable spray technology, the mainstream variable spray technology mainly includes pressure regulation, concentration regulation, and PWM regulation. In contrast, the pressure control type has a small flow adjustment range, easy distortion of spray characteristics, and the response speed of the concentration control type system is slow, which cannot meet the needs of real-time precision spraying, while the PWM control type system adjusts the spray flow of the nozzle by changing the operating frequency and duty cycle of the solenoid valve. It has the advantages of good dynamic characteristics, high control accuracy, fast system response, and a large flow adjustment range [27]. Therefore, this study decided to adopt PWM-regulated variable spray technology. The variable weeding control system designed in this study is composed of three subsystems, namely, the imaging subsystem, remote host computer, and variable spraying control subsystem. The specific structure of the system is shown in Figure 1.

The variable spray control subsystem is powered by a lithium battery (24 V–13 Ah, VOA, Huizhou, China). The system uses a DC 24 V vacuum diaphragm pump (A42-24, AUSEANA, Ningde, China) to meet the system’s need for a compact, lightweight design. To ensure proper operation of the system, the diaphragm pump is fitted with a filter on the water inlet to filter out large impurities from the liquid. The filter inlet is closely connected to the pesticide tank through a water pipe. On top of the pesticide tank, an ultrasonic level sensor (MTK-ZPM, MEACON, Hangzhou, China) is installed to monitor the remaining liquid level of the pesticide tank in real time.

The outlet of the diaphragm pump is connected to two sub-pipes; one is the output pipe for spraying vegetation, and the other is the return pipe for controlling the water pressure. During system operation, the controller reads the pipe pressure value in real time from a pressure sensor (CKHTP-01, CHEKON, Shanghai, China) installed in the output pipe. Subsequently, the controller will calculate the corresponding output according to the algorithm of the system controller, and then control the opening and closing degrees of the proportional control valve (FRSQT11F-16P, FREESUN, Suzhou, China). Simply put, controlling the degree of opening and closing is controlling the amount of water returning to the return line, thus ensuring that the pressure in the output line remains constant.

In addition, a flow sensor (LWGY, WEIERTAI, Shanghai, China) was installed in the middle of the output pipe of the system. The controller detects the real-time flow rate of the system by capturing the number of signal pulses from the flow sensor. For spraying control, the system uses a combination of hollow cone nozzles (KZ80-16, LICHENG, China) and high-velocity solenoid valves (6013, Burkert, Baden-Wurttemberg, Germany). The controller uses pulse signals to control the opening and closing of the solenoid valves, thus accomplishing precise control of the spraying volume. This design makes the whole spraying process both efficient and accurate. 

### 2.2. Design of Spraying Controller

**Figure 2:**
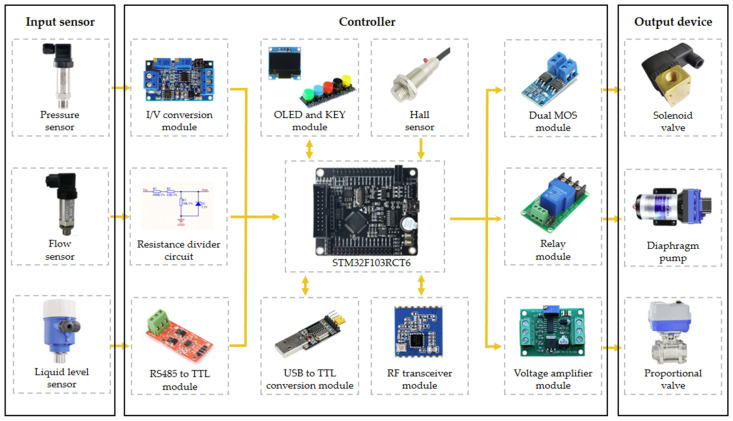
Electronic hardware design of the controller.

The variable spraying controller is designed based on a microcontroller (STM32F103RCT6, ST Microelectronics, Geneva, Switzerland) and the electronic hardware design scheme of the controller is shown in Figure 2. The output signal of the pressure sensor is a 4~20 ma current signal, which is converted into a 0~3.3 V voltage signal using an I/V converter circuit, and the microcontroller then reads the water pressure signal through an ADC converter. The microcontroller reads the pulse through the input capture function to achieve real-time detection of the spraying flow rate. The flow sensor output signal is a 24 V square wave pulse signal, and the accumulated 8500 pulses are equal to the flow rate of 1 L using the resistance divider circuit to convert the 24 V pulse signal to a 3.3 V pulse signal. The level sensor based on ultrasonic technology adopts the RS485 communication method, and the communication protocol is based on the Modbus protocol. The microcontroller reads the level information of the tank through TTL to the RS485 module (MAX3487, Analog Devices, Norwood, MA, USA). The microcontroller calculates the real-time traveling speed of the UGV by capturing the pulse output from the Hall sensor (JK8002D, JCXD, Nanjing, China) and adding them up, and the response frequency of this sensor is 100 KHZ. The microcomputer collects the real-time distribution of vegetation with the use of the camera and then relies on the algorithm to analyze the data in-depth and generate the corresponding intelligent decision making, and finally sends the decision-making data to the microcomputer through the USB to the TTL converter module. Finally, the decision data are sent to the controller through the USB to the TTL conversion module to achieve automated management. The RF-based wireless transceiver module (A7139, AMICCOM, Taiwan, China) is used for remote communication between the host computer and the controller, and sends the flow, pressure, liquid level, and operation status collected by the microcontroller to the host computer. A7139 is a wireless transceiver chip in the ISM band, with a maximum output power of up to 20 dBm, a maximum transmission rate of 250 Kbps, and a communication distance of up to more than 1 km in the open field. The communication distance in open space is up to more than 1 km, and there will be no network delay similar to that of a 4 G module, which is very suitable for the field of intelligent agriculture. The solenoid valve control circuit is a drive circuit using dual MOS tubes (AOD4180, AOS, San Francisco, CA, USA) in parallel. The advantages of MOS tubes include fast switching speed, lower power consumption, and strong anti-interference, and the dual MOS tube drive scheme can pass a larger current, which can improve the stability of the relay control circuit. The controller amplifies the 0~3.3 V signal output from the microcontroller DAC into 0~10 V signal through the voltage amplification module. Then, after being connected by the RVVP shielded cable, it is finally used to control the opening and closing degree of the proportional valve. The diaphragm pump starts and stops instantly and generates a large current, so it adopts a high-power relay module, and the principle is that the microcontroller achieves the control of the diaphragm pump by controlling the relay. The OLED screen (Blue-1.3 Inch, RISYM, Shenzhen, China) displays the system’s current spraying-related information in real time, and the KEY module can control the system’s starting and stopping, which both increases the convenience of debugging the system in practical use. Both add to the ease of commissioning the system in practice.

### 2.3. The Adaptive Fuzzy PID Control Algorithm

In the variable spraying system, the stability of the pipeline water pressure is a key prerequisite for the realization of accurate variable spraying. PID is widely used in the field of automatic control algorithms, consisting of the proportional unit (P), the integral unit (I), and the differential unit (D), which can be based on the difference between the set value and the actual value of the system through the proportional, integral, differential adjustment of the three parameters, the three-parameter values often directly determine the control effect [28]. Whether the values of these three parameters are reasonable or not will often directly determine the control effect. 

However, in the variable weeding process, the system needs to adjust the spraying amount of each nozzle according to the distribution of the target vegetation, resulting in nonlinear pressure changes in the pipeline, and the ideal control effect cannot be achieved using the traditional PID. Therefore, the system needs to dynamically adjust the integrating parameters of the PID control algorithm according to the pressure changes to ensure that the pressure of the system remains stable. The fuzzy control algorithm is a control strategy based on fuzzy logic, which realizes the control of a complex system by blurring the input and output variables, establishing a fuzzy rule base and performing defuzzification and other steps. In a fuzzy control system, the input and output variables do not only depend on the system state itself, but are also affected by the external environment, measurement errors, and other factors, so they have a certain degree of uncertainty and fuzziness. Fuzzy control algorithms are designed to deal with this uncertainty and ambiguity, which can handle various complex systems including uncertainty factors, and correct and optimize the output values [28]. Ultimately, this study adopts the method of combining the fuzzy control algorithm with the PID control algorithm. 

#### 2.3.1. Design of the Controller Structure

**Figure 3:**
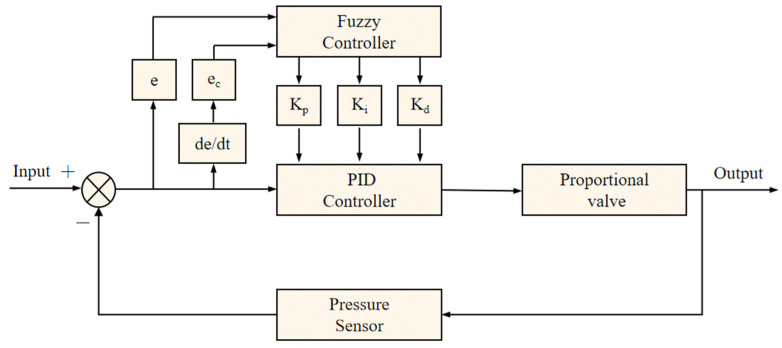
Block diagram of an adaptive fuzzy PID algorithm.

The adaptive fuzzy PID algorithm is an advanced control algorithm, which is mainly composed of a fuzzy controller and a PID controller. The structural design of the algorithm is shown in Figure 3. The core concept of the algorithm is to make full use of the advantages of the fuzzy controller and PID controller to realize the precise control of the water pressure of the output pipe [29].

During the operation of the algorithm, the algorithm reads the value of water pressure in the output pipe through the pressure sensor, and then compares this value with the preset value and calculates the error and the rate of change of the error. These calculated values are then fed into the PID controller to determine the control values used to regulate the state of the proportional valve and ultimately the output water pressure. The fuzzy controller is also involved in this process. It takes the error and the rate of change of the error as inputs and, using a set of predefined fuzzy rules, adaptively regulates the three regulation parameters: K_p_, K_i_, and K_d_. In this way, the system water pressure is maintained in a more stable state, both dynamically and statically. Compared with the traditional PID control algorithm, the adaptive fuzzy PID algorithm has greater flexibility and stability [29]. When dealing with non-linear sudden changes in water pressure, its advantages are particularly prominent. It can better adapt to a variety of complex situations and achieve more accurate and stable water pressure control. 

#### 2.3.2. Quantification of Input Values

The quantization of the input values is the projection of the input quantities e and e_c_ into a predefined corresponding domain using a quantization function, which is usually defined here as {−6, −5, −4, −3, −2, −1, 0, 1, 2, 3, 4, 5, 6} [30]. After taking readings of the water pressure variations during system operation, algorithms label the maximum pressure value read as Vmax and the minimum pressure value as V_min_, so that V_max_–V_min_ is the range of the deviation e, while the range of the deviation increment e_c_ is two times the range of the deviation e. The quantization function in this study has been adopted linearly. As such, its functional relationship is defined by Equation (1).

(1) $$\begin{array}{l} f\left(e\right)=\frac{6\;\times \;e}{V_{\max}\;-\;V_{\min}}\\ f\left(e_{c}\right)=\frac{6\;\times \;e_{c}}{2\left(V_{\max}\;-\;V_{\min}\right)} \end{array}$$

#### 2.3.3. Trigonometric Membership Function

**Figure 4:**
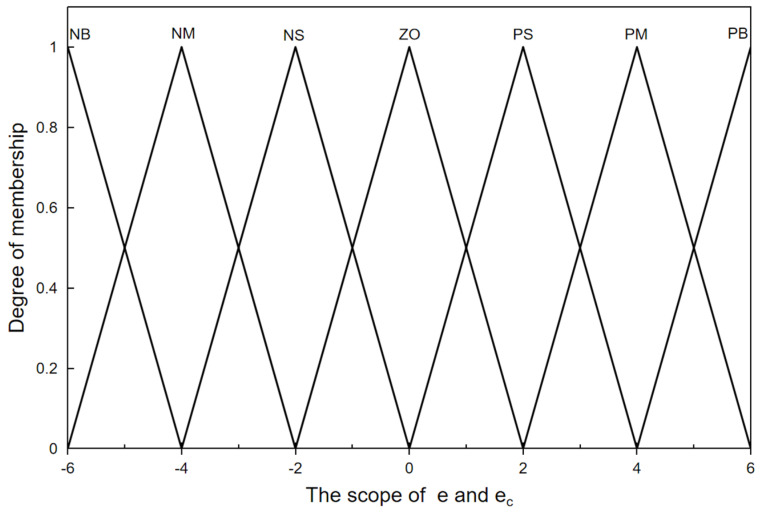
Trigonometric membership function for the input and output variables.

Firstly, the algorithm has to determine the fuzzy subsets of e and e_c_. In PID controllers, only seven linguistic variables, i.e., the negative big [NB], negative medium [NM], negative small [NS], zero [ZO], positive small [PS], positive medium [PM], and positive big [PB], are needed to be able to express their fuzzy subsets with sufficient accuracy. Therefore, the algorithm defines the fuzzy subsets of both e and e_c_ as {NB, NM, NS. ZO, PS, PM, PB} [30]. The degree of membership, which is a number between 0 and 1 that describes the degree to which the relevant input belongs to a fuzzy set, is then calculated by first determining the set to which e and e_c_ belong [30]. The triangular membership function is employed in this study to compute the membership of the input values, and Figure 4 illustrates how it works.

#### 2.3.4. Fuzzy Rules

The fuzzy rule base is the core of the fuzzy controller, which is established based on the fuzzification of the control quantities and relies heavily on the empirical knowledge of engineering experts to accomplish it. K_p_, K_i_, and K_d_ parameters are modified using the fuzzy rule basis, so it is necessary to establish the fuzzy rule base of these three parameters. According to the PID tuning experience, when the value of |e| is large, a larger value of K_p_ and a smaller value of K_d_ should be selected, which can improve the response speed of the system. When the value of |e| is medium, the value of K_p_ should be reduced, to reduce the overshoot of the system; the value of K_i_ should be selected as a moderate value. When the value of |e| is small, a larger value of K_p_ and K_i_ should be selected to avoid the system oscillating around the set value. When the value of |e_c_| is large, a smaller value of K_d_ should be selected. When the value of |e_c_| is large, a larger value of K_d_ should be selected [30]. Based on the above analysis, the fuzzy rules of K_p_, K_i_, and K_d_ are formulated in Table 1, Table 2 and Table 3.

#### 2.3.5. Decision Making Methods

The algorithm must go through defuzzification to achieve the intended result and get the right precise values. The final desired values in the adaptive fuzzy PID control are K_p_, K_i_, and K_d_, so the algorithm must also obtain the final desired values of K_p_, K_i_, and K_d_ based on the outcomes of the fuzzy reasoning. The inputs and outputs belong to the same affiliation at the same time because the inputs and outputs use the same thesis domain. After all, the algorithm employed the triangular affiliation function in the previous stage. This algorithm uses the center of gravity method to calculate the quantized values of the three output quantities by using Equation (2).

(2) $$\Delta K_{o}=\frac{\sum_{i=0}^{n}u_{c}\left(z_{i}\right)\cdot z_{i}}{\sum_{i=0}^{n}u_{c}\left(z_{i}\right)}$$

∆K_o_—the final output value of the fuzzy controller;
z_i_—the value in the fuzzy control volume domain;
u_c_(z_i_)—the degree of affiliation of z_i_.

After calculating the output of the fuzzy controller, the algorithm also introduces correction coefficients C_p_, C_i_, and C_d_ for adjusting the weights of the fuzzy controller outputs ∆K_p_, ∆K_i_, and ∆K_d_, respectively. Finally, after summing the fuzzy controller outputs with the K′_p_, K′_i_, and K′_d_ set after debugging for the original PID controller, respectively, the final three adjusted parameters of PID after adaptive fuzzy, K_p_, K_i_, and K_d_, are obtained. The specific functional relationship is defined by Equation (3).

(3) $$\begin{array}{l} K_{p}={K^{\prime }}_{p}+\Delta K_{p}*C_{p}\\ K_{i}={K^{\prime }}_{i}+\Delta K_{i}*C_{i}\\ K_{d}={K^{\prime }}_{d}+\Delta K_{d}*C_{d} \end{array}$$

### 2.4. Imaging Subsystem

Currently, for detecting the distribution of weeds, in addition to traditional methods of counting by hand or using local estimation techniques based on visual inspection by the human eye, modern technology offers many new options. Some of the more common methods are LiDAR, multispectral camera, and near-infrared camera. However, these sensors are invariably expensive, and their use and maintenance are more complex, requiring a certain level of professional knowledge and skills [31]. The principle and structure of visible light cameras are relatively simple, making them easy to use even for inexperienced users. Moreover, due to their relatively low manufacturing cost, they are easier to popularize and maintain. Therefore, for the industrialized application of this system in the future, this study adopts the approach of using visible light cameras to collect vegetation images.

**Figure 5:**
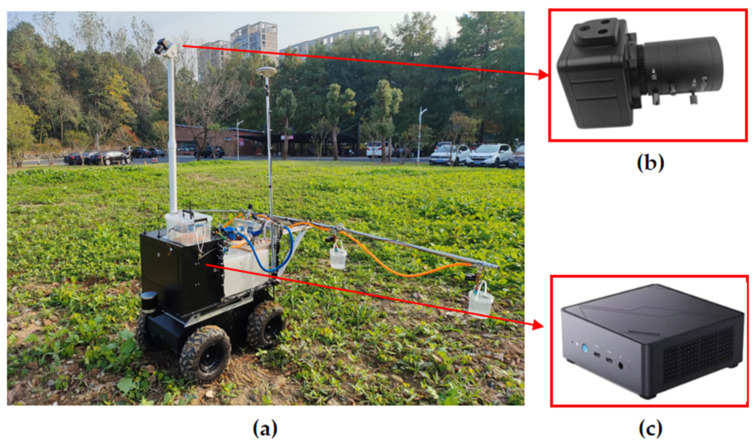
(**a**) The imaging sub-system on the UGV; (**b**) camera; (**c**) microcomputer.

The visible light camera is a 5-megapixel color drive-free industrial camera (CGU2-500C-UVC, CGimagetech, Hangzhou, China) with a USB 2.0 interface. In the experiment, the CMOS camera’s resolution was set to 1280 × 800 pixels. Since the degree of imaging distortion of the vegetation will directly affect the inspection results, an aberration-free lens was chosen. This type of lens typically adopts high-quality lenses and advanced optical technology, which can effectively inhibit the appearance of undesirable phenomena such as aberration and chromatic aberration, improve the clarity and fineness of the picture, and reduce the distortion and distortion of the edges of the picture. Therefore, when using this lens, there is no need for additional picture correction, which can greatly reduce the difficulty of post-processing and improve the accuracy of image algorithms. The focal length of the lens is 12 mm. The aperture range is F1.6-C and is manually adjustable, and the field of view (FOV) is 32°. The nozzles were mounted on the spray bar at the rear of the UGV (BullDog, YIKUN, Shanghai, China) as shown in Figure 5. Table 4 lists the detailed configurations of this UGV. In particular, the coverage of the spray application is related to the angle of the nozzle outlet, the mounting height, and the distance between the nozzles. To achieve the ideal coverage effect when other hardware devices are already installed, the spacing of each nozzle is reasonably adjusted in this paper, which effectively avoids overlapping or gaps in the spray coverage between nozzles. The camera was mounted above the front of the UGV, as shown in Figure 5, allowing for multiple rows of vegetation images to be captured simultaneously. The nozzle is mounted on the rear of the UGV, so there is a time delay between image acquisition and variable spraying, and the response time of the system needs to be controlled within this time delay. The vegetation images were transmitted by the camera to a microcomputer (S-1032, MECHREVO, Beijing, China) through a USB bus for processing, extracting tracts of information about weed characteristics, and calculating weed distribution. The nozzle is mounted on the rear of the UGV, resulting in a time delay between image acquisition and variable spraying. Controlling the response time of the system within this time delay is necessary. For this purpose, the camera transmits the vegetation images via a USB bus to a microcomputer, which processes the images, extracts information about weed characteristics, and calculates weed distribution. PyCharm Community Edition 2023 software was used as the development environment, with a series of customized algorithms developed using Python. The microcomputer is based on Intel’s I7-12650H processor, which has ten cores and 16 threads, with six performance cores and four energy-efficient cores. It has a maximum RWI of 4.7 GHz, a cache of 24 MB, and a TDP of 45 W. This processor offers excellent performance, low power consumption, and high maintainability, reducing repair and replacement costs. Finally, the microcomputer transmits the control information via a USB bus to the spray controller, which in turn controls the solenoid valve accordingly. 

#### 2.4.1. Graying of Images

A color image recorded by a visible light camera has a vast quantity of information because each pixel is composed of a mix of red, green, and blue ratios and can include up to 16 million different sorts of information. When processing color photos directly, a significant amount of calculation is needed, which might have a negative impact on operation speed. When performing image recognition, it is often enough to use only the information in the grayscale image, so this study chose to convert the three-channel color image into a single-channel grayscale map, which is more convenient for the algorithms to process it based on reducing the size of the image. The goal of image grayscaling is to speed up computation because grayscale images contain less information. Furthermore, compared to color photographs, grayscale images are simpler to process and feature-extract [31]. A commonly used grayscale processing method is the maximum value method.

#### 2.4.2. Segmentation of Images

In the fields of image processing and computer vision, image thresholding is a commonly used segmentation approach. Its primary purpose is to split an image into two or more sections based on the pixel values, which typically correspond to the target and background to be retrieved. When the background or the object has a single grayscale and the target and background have notable differences in grayscale characteristics, this segmentation method works especially well. The image is divided into target and background regions by selecting the appropriate threshold value. The primary benefits of threshold segmentation are its efficiency and simplicity [32]. Since it relies on the pixel value of a one-dimensional decision, it requires very little computation and can handle large amounts of image data quickly. This makes it ideal for variable weeding systems that need fast reaction times.

Every algorithm has a unique set of uses and restrictions, with the demands upon it varying depending on the application scenario, some require high computational efficiency, while others call for high precision. The histogram bimodal thresholding technique (HBT), fast iterative thresholding algorithm (FIT), thresholding algorithm based on K-means clustering, and OTSU thresholding algorithm are some of the most popular image thresholding algorithms. The segmentation threshold is determined by the histogram bimodal thresholding algorithm, which utilizes the bimodal features of the image grayscale histogram. While the calculation is straightforward and does not involve a laborious iteration or optimization process, the technique might not be suitable for certain complex images. A fast iterative threshold segmentation algorithm is a method that finds the optimal threshold iteratively; its basic idea is to start from an initial threshold, undergo continuous iterative computation, gradually approach the optimal threshold, and realize the segmentation of the image. The threshold segmentation algorithm based on K-means clustering can adaptively determine the segmentation threshold, which is suitable for image segmentation tasks with complex backgrounds and noise. However, the processing speed of this method may be slow due to the high computational complexity of the clustering algorithm. The OTSU threshold segmentation algorithm is a method that automatically determines the optimal threshold value by selecting it to maximize the interclass variance between the pixels of the two classes. This is suitable for situations where the difference between the target and background in the image is not obvious and it is necessary to determine the optimal threshold value computationally, but the amount of computation is large. 

#### 2.4.3. Assessment of the Distribution of Vegetation

**Figure 6:**
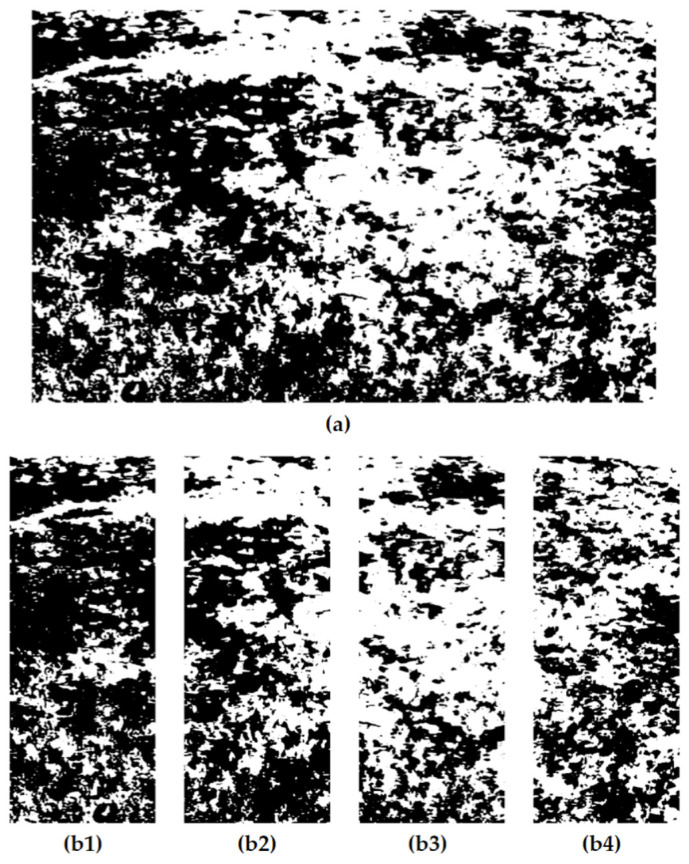
(**a**) The vegetation distribution in the field; (**b1**) the vegetation distribution in Plot 1; (**b2**) the vegetation distribution in Plot 2; (**b3**) the vegetation distribution in Plot 3; (**b4**) the vegetation distribution in Plot 4.

After the above steps, the imaging subsystem has successfully segmented the image into two regions, the target, and the background, and the final processing of the image using the vegetation distribution evaluation algorithm is still required. Firstly, the image segmentation algorithm will divide the vegetation image uniformly into four small farm images, each of which corresponds to a specific nozzle operation plot in the field, as shown in Figure 6. 

The imaging sub-system then extracts white pixels representing vegetation features from each plot and calculates the percentage of white pixels in each plot in the field using Equation (4), and these data allow for an accurate assessment of the distribution of vegetation in the field.

(4) $$P=\frac{sum\left(White\;pixel\right)}{Total\;pixel}\times 100\%$$

Finally, the system calculates a scientific reference amount of herbicide based on the percentage of vegetation distribution and then uses a PWM control method to accurately regulate the amount of herbicide sprayed from each nozzle to ensure optimal weed control. Figure 6 shows the distribution percentages of vegetation in each plot, which are 28.79%, 45.68%, 63.52%, and 52.12%, respectively.

## 3. Results

In the following experiments, after testing the performance of this vacuum diaphragm pump in this study, the operating water pressure of the variable mowing system was set to 0.2 MPA.

### 3.1. Adaptive Fuzzy PID Control

**Figure 7:**
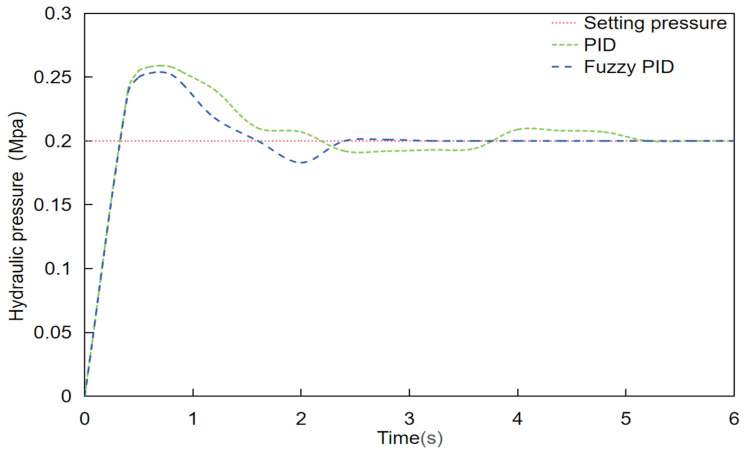
The conventional PID and the adaptive fuzzy PID.

In this experiment, to record the real-time water pressure changes of the system, a set of test upper computer software (PyCharm Community Edition 2023.2) based on the tkinter GUI library were developed. The software establishes a full-duplex communication mode with the system via a USB cable to ensure real-time and efficient data transmission. The upper computer can receive and display the water pressure data of the system in real time, and can graphically display the real-time changes in water pressure, providing researchers with an intuitive basis for data analysis. Before the start of the test, the tester set the control water pressure of the variable spraying system to 0.2 MPA, and tested the changes in the waveform of the start-up water pressure of the system under traditional PID control and adaptive fuzzy PID control, respectively. The results are shown in the accompanying Figure 7, which demonstrates the difference between the two. The test data show that the adaptive fuzzy PID control algorithm regulates the water pressure faster and the relative control accuracy is higher compared to the traditional PID control algorithm. In variable spraying scenarios, the control system’s adjustment speed and control accuracy of water pressure are crucial because the spraying amount needs to be adjusted frequently to adapt to the distribution of different vegetation. The adaptive fuzzy PID control algorithm is more suited for use in changeable spraying scenarios because it can better adjust to this demand and allow the spraying system to run steadily under these conditions. 

### 3.2. Staggered-Period PWM

**Figure 8:**
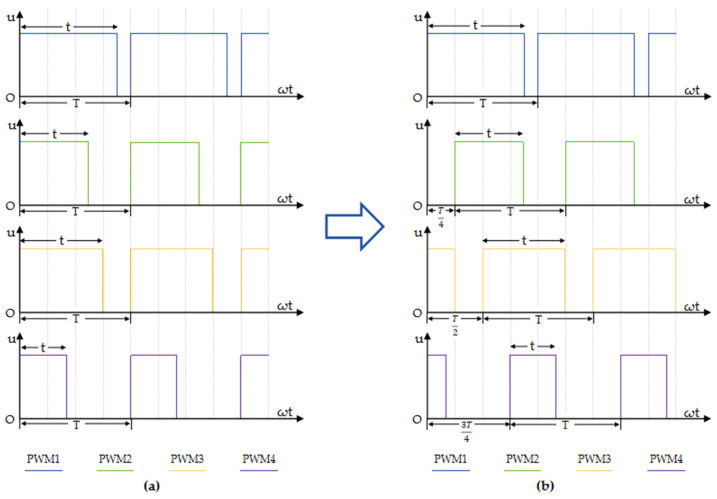
(**a**) Traditional PWM pulse waveform; (**b**) optimal PWM pulse waveform.

PWM (pulse width modulation) control technology is crucial for the automatic control domain. The idea is to use digital methods to regulate analog circuits, which is a way of digitally encoding analog signal levels and can dramatically lower system costs and power usage. The solenoid valve closure and disconnection are controlled using PWM technology in a variable spraying system, which allows the amount of spraying to be adjusted [33]. First, the control period is set to 3 HZ after consulting the solenoid valves’ minimum switching times. But there is an issue when controlling the system’s solenoid valves, as the water pressure in the pipeline will significantly fluctuate if all of the solenoid valves in the system are opened or closed at the same time and for the same duration, which will not only influence the precision of the spraying, but also has a significant pressure impact on the sensors and solenoid valves. Therefore, to reduce the large fluctuations in pipeline pressure caused by the overlap of the pulse period of the PWM control algorithm, this study designs a staggered-period PWM control method, as shown in Figure 8 (T is the control period and t is the on-time). 

To effectively reduce the drastic fluctuation of pipeline pressure triggered by the overlapping of pulse cycles in the PWM control algorithm, this study creates a staggered-cycle PWM control strategy. As shown in Figure 8 (where T represents the control period and t represents the conduction time), the core idea of this strategy is to stagger the control periods of the four solenoid valves by a time interval of T/4 in sequence. Through this careful timing arrangement, the four-solenoid valve opening and closing time can stagger each other, thus significantly reducing the overlap of the solenoid valve action time in the same control cycle. This design not only effectively suppresses large fluctuations in water pressure inside the pipeline and provides reliable protection for each sensor, but also further improves the accuracy and stability of variable spraying.

### 3.3. Spray Volume and Duty Cycle

**Figure 9:**
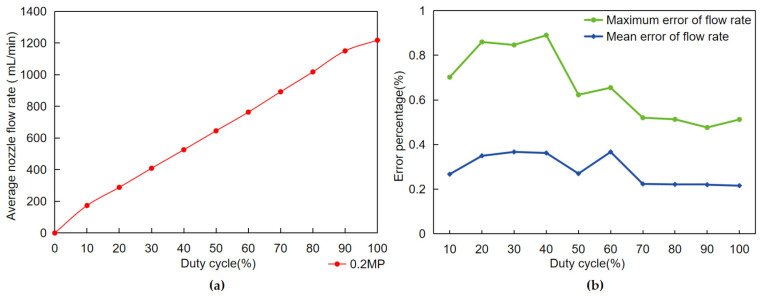
(**a**) Flow rate and duty cycle; (**b**) error rates for each duty cycle.

In this experiment, nozzles #2 to #4 were set to spray continuously at a 100% duty cycle, and then the PWM (pulse width modulation) duty cycle of nozzle #1 was varied in groups at 10% intervals for testing. In each group, nozzle #1 was used 10 times for 30 s and a measuring cup was used to collect the fluid from nozzle #1. Finally, the tester weighed the collected liquid on an electronic scale with an accuracy of 0.1 g and converted the unit of measurement to liters (L). Figure 9a shows the test results. According to the test results, the spray volume obtained shows a good linear relationship with the duty cycle of the PWM control signal. The matched spray volume increases with the gradual increase in the duty cycle of the PWM control signal. This finding provides the system with a direct and effective method for adjusting the spray volume, i.e., precisely controlling the spray volume of the nozzle by varying the duty cycle of the PWM signal. In a variable spray system, this linear relationship ensures that the system obtains the desired spray volume, thus achieving the goal of precise spraying.

With the spraying data obtained from the above tests, this paper then investigates the spraying stability of the variable spraying data. The results of the data analysis are shown in Figure 9b. The test results show that under all possible PWM duty cycle controls, the average error of variable spray for all nozzles is less than 0.5%, and the maximum error of variable spray for all nozzles is less than 1%. The test proved that this variable spray system has a very high accuracy when only a single nozzle is adjusted.

### 3.4. Orthogonal Tests of the System

In the actual spraying process, different spraying totals affect the accuracy of the spray. When the total amount of spraying in the system decreases, the spraying accuracy tends to decrease; conversely, when the total amount of spray in the system increases, the spraying accuracy tends to increase. To investigate this issue in depth, an orthogonal test was designed in this study to analyze the effect of different duty cycle combinations on spraying accuracy. The orthogonal test is a commonly used experimental design method that can effectively reduce the number of experiments while ensuring the accuracy and reliability of the experiment. This method is based on the Galois theory, and some representative duty cycle combinations are selected from the full-scale experiments. Given that the system’s overall spraying amount cannot be less than 25% in this study, four typical spraying duty cycles—25%, 50%, 75%, and 100%—were chosen to create an orthogonal Table 5. The assessment of spraying accuracy in this trial continued in the same way as in the previous trial.

**Figure 10:**
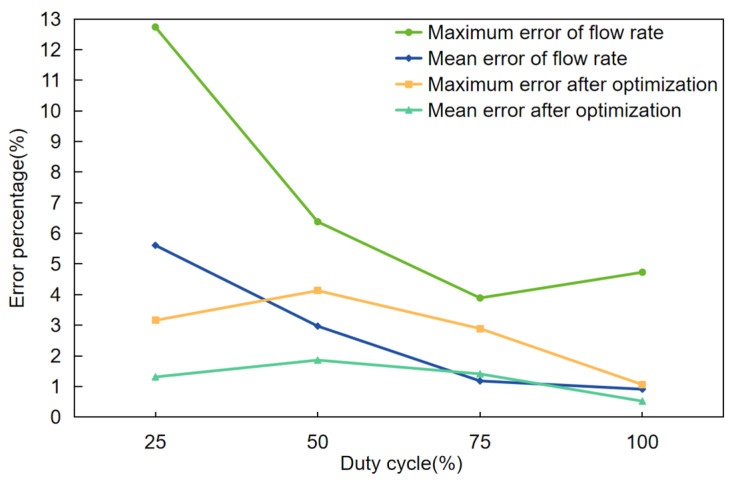
Error rates for four duty cycles after optimizing the strategy.

The orthogonal tests described above allow us to derive a more comprehensive and representative picture of the spraying accuracy of each nozzle under different duty cycle combinations. To further understand the characteristics of these data, an in-depth error analysis was conducted for each set of data, with the main objective of ensuring the accuracy and reliability of the variable weed control system by analyzing the spraying error rate. The findings demonstrated that the duty cycle data at 50%, 75%, and 100% all partially satisfied the system’s expectations. One set of data, the 25% duty cycle data, however, had a significant mistake: the maximum error rate surpassed 12%, and the average error rate over 5%, as shown in Figure 10. Consequently, to determine the cause of this inaccuracy, it is critical to investigate and comprehend it in greater detail.

After an in-depth study of the data with a duty cycle of 25%, this study finds that the error rate increases linearly when the system is set to spray a total amount less than or equal to 50% of the total amount of spray. In this case, the largest error is for the data of test number 1 (25%, 25%, 25%, 25%), which obtains a spray volume 12% higher than the expected spray volume. After several subsequent repetitions of the test, it was found that when the total spray volume was fixed, the degree of variation in the error values produced was minimal. This indicates that the error rate generated by the system is stable when the total spraying volume is constant. After further observation and validation of the orthogonal test data, this study also found that the spraying accuracy is also related to its control sequence. In this system, if the inner nozzle is energized first, the accuracy of the outer nozzle decreases; on the contrary, if the outer nozzle is energized first, the effect on the inner nozzle is less.

Finally, based on the above experiments, this paper adjusted and optimized the spraying control system strategy, which effectively reduces the impact of the control sequence between the nozzles, and real-time monitoring of the spraying flow condition through the algorithm to achieve automatic adjustment of the control signal. The results show that the average error rate of the 25% duty cycle data after optimization is less than 2%, and the maximum error rate is less than 5%, as shown in Figure 10. This series of improvement measures can ensure that all nozzles can be combined in a way that meets the system performance requirements while minimizing errors, thus guaranteeing the stability and accuracy of the system.

### 3.5. Experiments with Image Segmentation Algorithms

**Figure 11:**
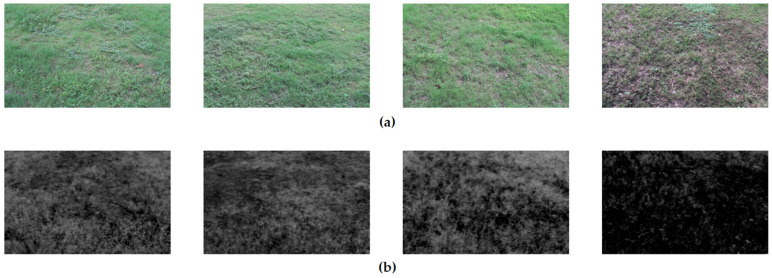
The image after grayscale: (**a**) RGB original image; (**b**) gray image.

After comparing various image greyscaling algorithms, this study finds that the weighted value method has significant advantages in processing green vegetation images. It can well suppress unnecessary image details such as shadows, dead grass, and soil, thus making the image features of vegetation more prominent. Based on this finding, this system organically combines the normalized super green algorithm (2G-R-B) with the threshold segmentation method, to effectively differentiate between the target and the background in the image, and further improve the accuracy and effect of image processing, as shown in Figure 11.

**Figure 12:**
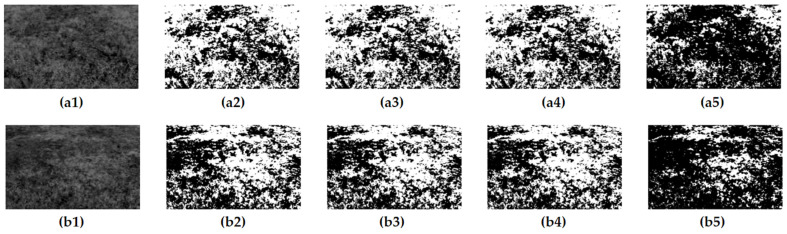
The image after threshold segmentation: (**a1**) gray image; (**a2**) OTSU; (**a3**) K-means; (**a4**) FIT; (**a5**) HBT; (**b1**) gray image; (**b2**) OTSU; (**b3**) K-means; (**b4**) FIT; (**b5**) HBT.

In this experiment, the tester carefully collected 40 representative vegetation images from field experiments as the test samples. To explore and validate the most suitable threshold segmentation algorithm, this paper applied the histogram bimodal threshold segmentation algorithm (HBT), the fast iterative threshold segmentation algorithm (FIT), the threshold segmentation algorithm based on K-means clustering, and the classical OTSU threshold segmentation algorithm to process these images in a comprehensive and detailed way. This process aims to reveal the advantages and limitations of various algorithms in processing complex vegetation images through comparative analysis, to provide strong algorithmic support and reference basis for our subsequent applied research. Figure 12 illustrates the segmentation results of two of the vegetation images and these results clearly show the differences and characteristics of the different algorithms in processing the vegetation images.

To evaluate the performance of various threshold segmentation algorithms more comprehensively, this paper uses the average threshold error Equation (5) and the pixel extraction accuracy Equation (6) to calculate the computation time, average threshold error, and average accuracy of each algorithm. The experimental results show that although the HBT algorithm can obtain better segmentation results, it loses some of the detailed information of the image and is therefore not ideal. In contrast, the FIT algorithm, the K-means algorithm, and the OTSU algorithm have almost the same better processing results and can accurately realize the segmentation of the image.

(5) $$\varepsilon =\frac{\sum_{i}^{N}\left|\left(TO_{i}-T_{i}\right)\right|}{N}$$

(6) $$\rho =\frac{\sum_{i}^{N}\left|\left(PO_{i}-P_{i}\right)\right|}{N}\times 100\%$$

where N is the total number of images (N = 40). T_i_ is the threshold for different algorithms and TO_i_ is the threshold for the OTSU method. P_i_ is the percentage distribution of vegetation for different algorithms calculated by Equation (6) above and PO_i_ is the percentage distribution of vegetation for the OTSU method. Table 6 lists the performance of the four segmentation algorithms, where the thresholds are averaged over 40 images.

The OTSU method can produce the best result, as Table 5 illustrates. However, to determine the optimal threshold, the algorithm must traverse all possible thresholds in the image and calculate the interclass variance between the pixels. As a result, real-time image processing is difficult to achieve and the computation process is time-consuming. The segmented image is not usable since it is unclear and lacks most of the detailed information, even if the HBT algorithm operates at the highest speed. Even though the K-means method’s operation time is much less than that of the OTSU algorithm, it still fails to meet the system’s requirements even if its findings are the closest to the ideal answer. In contrast, the FIT algorithm, although the final accuracy is 0.6% lower than the HBT algorithm, the operation speed is seven times faster than the HBT algorithm, and the operation time is very close to that of the HBT algorithm, but the accuracy of the segmentation results is much higher than that of the HBT algorithm. Considering the factors of segmentation accuracy and operation speed, this study finally chooses the FIT algorithm to segment the image.

### 3.6. Response Test of the System

**Figure 13:**
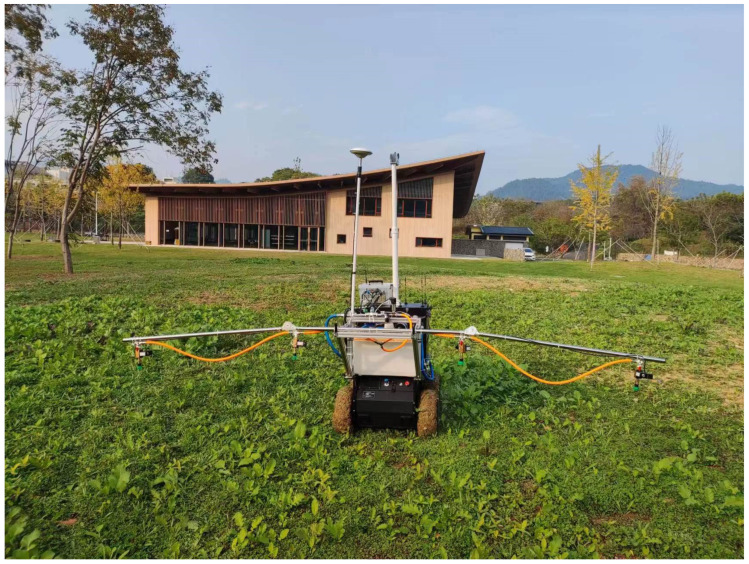
Evaluation of the variable weeding system.

In the field test, this study set the traveling speed of the UGV to 1 m/s. The distance from the camera to the nozzle is 1.2 m, which means that the system needs to strictly control the response time within 1.2 s from the beginning of image acquisition to the completion of variable spraying. This response process consists of five steps: reading the image, saving the image, processing the image, transmitting the data, and controlling the spraying. The mapping length of the image in the field has a decisive influence on the acquisition frequency of the system. To indirectly control the acquisition frequency of the system, the camera is adjusted by changing the angle of the camera mounted in front of the UGV. To minimize the proportional distortion of the images, the length of the acquired images was set in this study to be the image at a distance of 1~3 m from the farmland in front of the UGV, which resulted in an image acquisition interval of 2 s for the system. The evaluation UGV is shown in Figure 13. 

This experiment was conducted with the help of several techniques and instruments in order to accurately record the response time of the system. Firstly, this paper calculates the time spent on each image processing by means of algorithmic de-recording timestamps, which represent the algorithm’s performance metrics in terms of data processing. Then, the time spent by the controller from accepting data via DMA (direct memory access) to completing the PWM (pulse width modulation) control, which is the hardware runtime, is recorded by using an oscilloscope to capture the pulses. Accurate measurements of these two time periods provide a comprehensive understanding of the actual response time of the system, thus providing an important reference for subsequent system optimization and improvement.

This study had a total of thirty trials, and the average response time for the imaging subsystem is 0.0345 s, as indicated in Table 7. The command period of the controller (STM32F103RCT6) is about 0.0174 us, and the average response time of the spraying controller from receiving the control command to issuing the PWM control signal is about 0.0137 s. The PWM signal is used to control the solenoid valve, which enables the function of regulating the spraying amount. Therefore, the response time of the whole system is about 0.0482 s (0.0345 s + 0.0137 s), which can satisfy the requirement of real-time processing.

Although the UGV can use its supporting WEB terminal to set the traveling speed, in the actual operation, there are many unpredictable factors, such as slope, soil, vegetation, and humidity, which may interfere with the traveling speed set by the UGV. Due to the variation in the speed of the UGV, the images captured by the system may have discontinuous problems, which can lead to the overlap or omission of the articulated part between the front and back pictures. This situation will directly affect the precise spraying effect on the whole area of the farmland.

To further ensure the stability of spraying, the system obtains the current driving speed of the UGV through the Hall sensor. The Hall sensor is a dependable technique for measuring speed since it can record the UGV’s speed in real-time and send the information back to the control system. When there is a large deviation between the traveling speed and the set speed, the system will adjust accordingly to the real-time traveling speed of the UGV. This method not only ensures the continuity and stability of the spraying operation but also realizes the application of the system on different types of carriers, thus providing a more flexible and efficient solution for various agricultural scenarios.

## 4. Conclusions

The main conclusions of this study are as follows:

(1) A variable spraying and weeding system based on STM32 is developed. In this paper, a low-cost and high-efficiency constant pressure control scheme is innovatively proposed by utilizing the powerful processing capability and excellent real-time performance of STM32. The core principle is to control the degree of opening and closing of the high-speed proportional control valve through STM32 to realize the adjustment of the flow rate of the return pipe, and then complete the stable pressure control of the whole pipeline system. The program effectively overcomes the shortcomings of the traditional controller, not only significantly improving the control accuracy, but more importantly, greatly reducing the cost of the traditional constant pressure control program.
(2) The system utilizes fuzzy rules to achieve adaptive modification of the K_p_, K_i_, and K_d_ adjustment parameters of the PID control algorithm and combines them with an interleaved period PWM controller to reduce the impact of nonlinear variations in water pressure on the performance of the system, and to improve the stability and control accuracy of the system. The test results show that during the test, the pressure variation is less than ±0.05 MPa, the average error of spraying is less than 2%, and the maximum error is less than 5%. The improvement of spraying accuracy and spraying stability of the variable spraying system was accomplished. In conclusion, this study verifies that the adaptive fuzzy PID algorithm has superior performance in variable spraying systems by comparing the effects of traditional PID control and adaptive fuzzy PID control in practical applications.
(3) In this study, after comparing and testing a variety of image graying algorithms and image threshold segmentation algorithms, the combination of the normalized super green algorithm (2G-R-B) and the FIT threshold segmentation method was finally adopted, which efficiently and quickly distinguished between vegetation and background. Finally, the performance of the system was comprehensively evaluated through field trials. The evaluation showed that the system was able to adjust the corresponding spraying volume in real-time according to the vegetation distribution under the decision-making based on machine vision algorithms, which proved the effectiveness of the designed variable weed control system. Compared with other vegetation image detection techniques, it solves the problem that real-time on-demand spraying is not possible due to the slow response time of detection.
(4) In this study, the variable weeding system is coupled with the autonomous path planning function of a UGV to successfully realize unmanned variable weeding operations, which enables the personnel concerned to be freed from the heavy weeding work and greatly improves operational efficiency and safety. In addition, this system is not only extremely costly, but also highly adaptable, making it very suitable for use with unmanned aircraft, and traditional agricultural equipment, and also can be used with various types of vehicles. Its characteristics give the system great potential for industrialization and create favorable conditions for the marketing of the variable weeding system. Therefore, the variable weeding system developed in this study has high application value and is of far-reaching significance in promoting the future development of agriculture in the direction of intelligence, refinement, and efficiency.

## Figures and Tables

**Table 1:** Fuzzy control rules of ∆K_p_.

∆K_p_		e_c_						
		NB	NM	NS	ZO	PS	PM	PB
e	NB	PB	PB	PM	PM	PS	ZO	ZO
	NM	PB	PB	PM	PS	PS	ZO	NS
	NS	PM	PM	PM	PS	ZO	NS	NS
	ZO	PM	PM	PS	ZO	NS	NM	NM
	PS	PS	PS	ZO	NS	NS	NM	NM
	PM	PS	ZO	NS	NM	NM	NM	NB
	PB	ZO	ZO	NM	NM	NM	NB	NB

**Table 2:** Fuzzy control rules of ∆K_i_.

∆K_i_		e_c_						
		NB	NM	NS	ZO	PS	PM	PB
e	NB	NB	NB	NM	NM	NS	ZO	ZO
	NM	NB	NB	NM	NS	NS	ZO	ZO
	NS	NB	NM	NS	NS	ZO	PS	PS
	ZO	NM	NM	NS	ZO	PS	PM	PM
	PS	NM	NS	ZO	PS	PM	PM	PB
	PM	ZO	ZO	PS	PS	PM	PB	PB
	PB	ZO	ZO	PS	PM	PM	PB	PB

**Table 3:** Fuzzy control rules of ∆K_d_.

∆K_d_		e_c_						
		NB	NM	NS	ZO	PS	PM	PB
e	NB	PS	NS	NB	NB	NB	NM	PS
	NM	PS	NS	NB	NM	NM	NS	ZO
	NS	ZO	NS	NM	NM	NS	NS	ZO
	ZO	ZO	NS	NS	NS	NS	NS	ZO
	PS	ZO	ZO	ZO	ZO	ZO	ZO	ZO
	PM	PB	NS	PS	PS	PS	PS	PB
	PB	PB	PM	PM	PM	PS	PS	PB

**Table 4:** UGV details.

Specification	
**Model**	BullDog
Manufacturer	Shanghai Yikun Electrical Engineering Co., Ltd, Shanghai, China.
Weight	100 kg
Size	914 × 708 × 440 mm
Maximum load	100 kg
Maximum speed	2.0 m/s
API	ROS/Python/C++
Working hours	3 h typ/8 h max
Power	2 × 1000 W Peak
Encoder	2000 Wire/turn
Control mode	Current, Speed, Wheel speed
Communication	USB/Ethernet
Battery type	48 V 20 Ah lithium battery
Level of protection	IP66
Maximum climbing angle	40°

**Table 5:** Table of orthogonal experimental designs.

Test Number	Column Number			
	A	B	C	D
	Duty Cycle (%)	Duty Cycle (%)	Duty Cycle (%)	Duty Cycle (%)
1	25	25	25	25
2	25	50	50	50
3	25	75	75	75
4	25	100	100	100
5	50	25	50	75
6	50	50	25	100
7	50	75	100	25
8	50	100	75	50
9	75	25	75	100
10	75	50	100	75
11	75	75	25	50
12	75	100	50	25
13	100	25	100	50
14	100	50	75	25
15	100	75	50	100
16	100	100	25	75

**Table 6:** Evaluation of the performance of threshold segmentation algorithms.

Algorithm	Image Size	Running Time (s)	Threshold Value	Threshold Error	Precision Rate (%)
OTSU	1280 × 800	0.2511	62.75	0	100
K-means	1280 × 800	0.1770	62.92	0.3281	99.6068
FIT	1280 × 800	0.0252	63.65	0.9203	99.0143
HBT	1280 × 800	0.0116	81.00	18.3500	76.3109

**Table 7:** Tests of system response time.

Test	Response Time (s)					Mean Response Time (s)
	1	2	3	4	5	
1	0.0410	0.0347	0.0411	0.0340	0.0351	0.0371
2	0.0388	0.0384	0.0351	0.0320	0.0356	0.0359
3	0.0295	0.0286	0.0294	0.0404	0.0310	0.0317
4	0.0300	0.0375	0.0419	0.0338	0.0447	0.0375
5	0.0286	0.4234	0.0412	0.0372	0.0248	0.0348
6	0.0307	0.0376	0.0349	0.0330	0.0314	0.0300

## Data Availability

Data are contained within the article.
